# Nimbolide: promising agent for prevention and treatment of chronic diseases (recent update)

**DOI:** 10.29219/fnr.v68.9650

**Published:** 2024-03-18

**Authors:** Peramaiyan Rajendran, Kaviyarasi Renu, Basem M. Abdallah, Enas M. Ali, Vishnu Priya Veeraraghavan, Kalaiselvi Sivalingam, Yashika Rustagi, Salaheldin Abdelraouf Abdelsalam, Rashid Ismael Hag Ibrahim, Saeed Yaseen Al-Ramadan

**Affiliations:** 1 Department of Biological Sciences, College of Science, King Faisal University, Al Ahsa, Saudi Arabia; 2 Centre of Molecular Medicine and Diagnostics (COMManD), Department of Biochemistry, Saveetha Dental College & Hospitals, Saveetha Institute of Medical and Technical Sciences, Saveetha University, Chennai, Tamil Nadu, India; 3 Department of Botany and Microbiology, Faculty of Science, Cairo University, Cairo, Egypt; 4 Department of Developmental, Molecular and Chemical Biology, Tufts University School of Medicine, Boston, MA, USA; 5 Centre for Cancer Genomics, Department of Medical Oncology, Dana-Farber Cancer Institute, Boston, MA, USA; 6 Department of Zoology, Faculty of Science, Assiut University, Assiut, Egypt; 7 Department of Botany, Faculty of Science, University of Khartoum, Sudan; 8 Department of Anatomy, College of Veterinary Medicine, King Faisal University, Al-Ahsa, Saudi Arabia

**Keywords:** chronic diseases, limonoids, Azadirachta indica, nimbolide, EMT, Wnt-β/catenin

## Abstract

**Background:**

Nimbolide, a bioactive compound derived from the neem tree, has garnered attention as a potential breakthrough in the prevention and treatment of chronic diseases. Recent updates in research highlight its multifaceted pharmacological properties, demonstrating anti-inflammatory, antioxidant, and anticancer effects. With a rich history in traditional medicine, nimbolide efficacy in addressing the molecular complexities of conditions such as cardiovascular diseases, diabetes, and cancer positions it as a promising candidate for further exploration. As studies progress, the recent update underscores the growing optimism surrounding nimbolide as a valuable tool in the ongoing pursuit of innovative therapeutic strategies for chronic diseases.

**Methods:**

The comprehensive search of the literature was done until September 2020 on the MEDLINE, Embase, Scopus and Web of Knowledge databases.

**Results:**

Most studies have shown the Nimbolide is one of the most potent limonoids derived from the flowers and leaves of neem (Azadirachta indica), which is widely used to treat a variety of human diseases. In chronic diseases, nimbolide reported to modulate the key signaling pathways, such as Mitogen-activated protein kinases (MAPKs), Wingless-related integration site-β (Wnt-β)/catenin, NF-κB, PI3K/AKT, and signaling molecules, such as transforming growth factor (TGF-β), Matrix metalloproteinases (MMPs), Vascular Endothelial Growth Factor (VEGF), inflammatory cytokines, and epithelial-mesenchymal transition (EMT) proteins. Nimbolide has anti-inflammatory, anti-microbial, and anti-cancer properties, which make it an intriguing compound for research. Nimbolide demonstrated therapeutic potential for osteoarthritis, rheumatoid arthritis, cardiovascular, inflammation and cancer.

**Conclusion:**

The current review mainly focused on understanding the molecular mechanisms underlying the therapecutic effects of nimbolide in chronic diseases.

## Popular scientific summary

- Nimbolide, obtained from neem (Azadirachta indica), is a highly strong limonoid found in the flowers and leaves of the plant.
- It is extensively utilized for the treatment of various human ailments.
- It has been found to regulate the primary signaling pathways.
- It possesses anti-microbial, anti-inflammatory, and anti-cancer characteristics, rendering it a captivating molecule for scientific investigation.
- Nimbolide exhibited therapeutic efficacy in the treatment of rheumatoid arthritis, inflammation, osteoarthritis, cardiovascular diseases and cancer.

Worldwide, chronic diseases account for the majority of morbidity and mortality, both in developed and developing countries. Cardiovascular diseases (CVDs), chronic respiratory diseases, cancer, and diabetes are all chronic diseases or non-communicable diseases (1). There are a lot of paths that are involved in these diseases (CVDs, chronic respiratory diseases, cancer, and diabetes) so focusing on just one of them might not solve the problem (2). There are a number of medicines for these long-term conditions, but most of them have side effects. Most of the time, the word ‘side effect’ refers to an undesirable effect that a drug or medication has in addition to its main effect. There may be different side effects for each person, based on their disease, weight, age, gender, and race. So, the present toxic and ineffective treatment plans need to be replaced with less expensive, safer, more effective, and multi-targeted plans. There is a lot of hope that medicinal plants can avoid cancer and other long-term diseases (3, 4). Preclinical and clinical evidence suggests that phytochemicals can help to treat a wide range of chronic diseases, and different formulations can increase their availability (5, 6).

The Neem tree *Azadirachta indica* (also known as Melia azadirachta [L] and *Antelaeaazadirachta* [L]) is an oblique-leafed tropical evergreen tree with insect-repellent properties (7). The region formerly known as Myanmar, which encompasses India as well as other nations such as Bangladesh, Sri Lanka, and parts of Africa, is the subject of discussion. In the region of East Africa, there exists a medicinal plant commonly referred to as Mwarobaini in the Swahili language. This plant has been traditionally employed for the treatment of a diverse range of 40 different ailments (8–10).

**Fig. 1:**
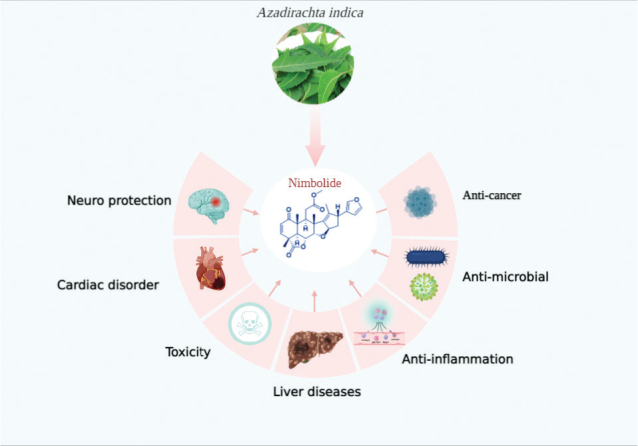
The use of nimbolide as well as its metabolites in the treatment of chronic diseases. (Image created from biorender.com).

There are many domestic, industrial, and pharmaceutical values in the Neem tree. Foliar extracts of this plant are used as a panacea for skin problems such as eczema, ringworm, acne and cancer (27). Neem tree is highly rich in medicinal bioactive compounds including mainly the tetranortriterpenoids or limonoids. Nimbolide is one of the tetranortriterpenoid with several pharmacological properties including antimalaria, antibacterial, antiviral, antioxidative, anti-inflammatory, antiinvasive, neuroprotective, hepatoprotective, and pro-apoptotic properties (Table 1) (27, 28). For example, the antioxidant nimbolide was found to be more potent than azadirachtin and ascorbic acid. Nimbolide is an active ingredient in neem extract, an Indian Ayurvedic herb used for acne, wounds, gastric ulcers, and infections (29). Nimbolide was shown to be the most potent cytotoxic among six limonoids (30). Various molecular targets have been identified through investigation of nimblolide’s action in multiple cancer cell lines (31). There are a bunch of studies showing that nimbolide (Fig. 1).

**Table 1:** Nimbolide biological effects various organs

	Organs	Biological activities	Mode of study	Target	References
1	Liver	**Protective Effect** Hydroxyurea induced toxicity High fat induced obesity CCl4 induced toxicity Streptozotocin induced diabetic Diabetic wound healing activity	In vivo In vivo In vivo In vivo In vivo In vivo	Inhibit oxidative stress Activate Nrf2/HO-1 signaling Inhibit oxidative stress Anti-inflammatory and antioxidant TLR4/NF-κB signaling pathway Anti-inflammatory and antioxidant	(11–17)
2	Heart	Doxorubicin induced toxicity High fat induced toxicity	In vivo In vivo	PI3K/AKT and NF-kB signaling Down regulation of lipid profile	(18–21)
4	Brain	Neuro inflammation Alzheimer’s disease Neurodegenerative	In vitro In vivo Molecular docking	p38 and JNK MAPKs down regulation of AChE and Aβ GSK-3β interaction	(22–26)

## Nimbolide chemical structural

Nimbolide systematic name: methyl 2-[(1R,2S,4R,6R,9R, 10S,11R,15R,18R)-6-(furan-3-yl)-7,9,11,15-tetramethyl-12,16-dioxo-3,17dioxapentacyclo[9.6.1.02,9.04,8.015,18]octadeca-7,13-dien-10-yl]acetate], formula: C27H30O7, molecular weight: 466.5 (PubChem CID: 12313376). Nimbolide extracted from the leaves of *Azadirachta indica* is known as nimbolide (I), a tetranortriterpenoid. In the presence of tetrabutyl ammonium bromide, nimbolide was rearranged with the intent of enhancing the native compound’s activity. The chemical modification separated the isotopes C7 and C15. The double bond in isonimbolide is rearranged from C13 = C14 in nimbolide to C16 = C17 in isonimbolide, with a ring closure of C7 and C13, resulting in the reformation of the ether linkage (32).

The ether linkage in nimbolide is between C7 and C15, while in isonimbolide it’s between C7 and C13. As a result, the main difference between nimbolide and isonimbolide is the orientation of the ─CH_2_COOMe group attached to C9. In addition, an isonimbolide has a torsion angle of 132.5 (6) and a nimbolide has 91.1 (5, 32).

## Nimbolide molecular targets

Numerous studies have demonstrated the exceptional ability of nimbolide to prevent and treat severe chronic diseases, such as inflammatory diseases, neurological diseases, CVDs, cancer, diabetes, etc., by modulating multiple pathways (33). In acute respiratory distress syndrome (ARDS), nimbolide reported to significantly inhibit nitrosative-oxidative stress, managed inflammatory cytokines, and iNOS, myeloperoxidase, and nitro tyrosine levels (34). In ARDS, nimbolide inhibits specifically both TNF-α and NF-κB, and activates the anti-inflammatory mechanism (35). Along with that the mechanism of nimbolide cytotoxicity in various pathologies has different functions, including the function of nimbolide as a modulator of the macrophage migration inhibitory factor. Consequently, nimbolide inhibits the ability of NF-kβ to translocate and thus, preventing the release of pro-inflammatory cytokines such as TNF-α, IL-1α, IL-1β, and IL-6 (17, 36–38).

**Fig. 2:**
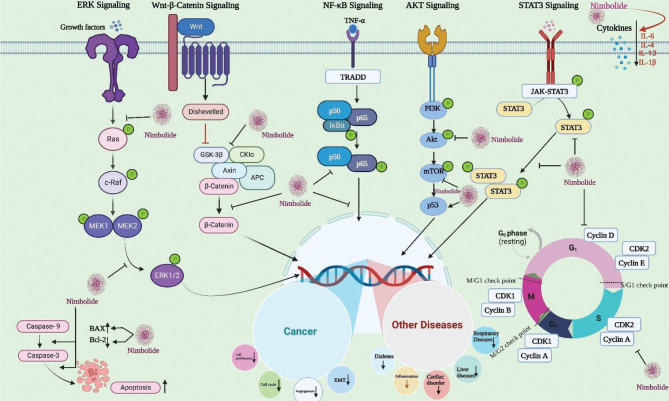
An overview of the mode of action of nimbolide in various chronic diseases treatment (Image created from biorender.com).

A study by Alshammari et al., and coworkers studied the effect of nimbolide on intracellular lipid deposition and oxidative stress in primary hepatocytes induced by oleic acid supplementation for 24 h. Nimbolide reduced intracellular cholesterol, free fatty acids, and triglycerides and enhanced hepatocyte function by inhibiting oxidative DNA damage and lipid peroxidation through its antioxidant effects (21). Moreover, it improves endogenous antioxidant levels such as Glutathione (GSH) and antioxidant enzyme activities and restores mitochondrial potential. Nimbolide also showed a number of biological effects including inhibition of lipid peroxidation, DNA damage, hypolipidemic effects, reactive oxygen species (ROS) inhibition, increasing antioxidant enzyme activities and restoring mitochondrial function (39). These functions and mechanisms of action of nimbolide against various chronic diseases are mediated via regulating different signaling pathways, including the NF-κB, STAT3, Notch-1 pathways, PTEN/PI3K/AKT and ERK ½ pathways and their downstream targets LXRα, PPARγ (Fig. 2) (21, 31, 40–47).

Nimbolide showed immense antioxidant properties. Nimbolide prevented ccl4-induced lipid peroxidation (33), and protected AML12 cells from LPS-induced damage (42). Shu and co-workers demonstrated the potential of nimbolide to reduce food intake and blood glucose, and body weight in STZ-provoked animals. A nimbolide treatment also remarkably suppressed levels of MCP-1, VEGF, and MMP-9, pro-inflammatory mediators, cholesterol, TG, LDL, and HDL. Nimbolide significantly inhibited the TLR4/NF-B pathway (15, 48).

### Antimicrobial activity of nimbolide

Nimbolide showed high ability to inhibit a wide variety of microbes, including bacteria, pathogenic fungi and viruses. Nimbolide exhibits enhanced efficacy against microbes, increasing their susceptibility to its antimicrobial effects (49). Nimbolide exhibited activity against a vast number of bacterial species including *Streptococcus mutans*, *Enterococcus faecalis*, *Streptococcus mitis*, *Streptococcus salivarius*, streptomycin-resistant strains and even *Streptococcus sanguis* (49–56). Furthermore, nimbolide is effective against plaque and, showed to inhibit the formation of biofilms in case of *Pseudomonas aeruginosa* (57, 58). Nimbolide significantly blocked HSV-1 entry into cells and inhibited HSV-1 activity when pre-incubated with virus, but not with target cells, suggesting, that nimbolide has direct anti-HSV-1 properties (54). As shown by virus inactivation and yield reduction assays, nimbolide has virucidal activity against coxsackie virus virus B-4 (59). As an antifungal agent, nimbolide showed to significantly inhibited and controlled the growth of Aspergillus and Rhizopus.

## Anti-inflammatory effect of nimbolide in respiratory diseases

ARDS causes refractory hypoxemia due to an excessive acute inflammatory response in the lung parenchyma. One of the earliest abnormalities seen in the lung injury is the expression of soluble tumor necrosis factor (TNF-α), which exerts cytotoxicity in epithelial and endothelial cells further causes edema (60, 61). Pool and coworkers showed that nimbolide blocks chemokines, nitrosative-oxidative stress and inflammatory cytokines by suppressing iNOS, myeloperoxidase, and nitrotyrosine expression. Furthermore, nimbolide reduces neutrophil and mast cell migration while normalizing hypothermia caused by LPS. Nimbolide showed to modulate the expression of epigenetic regulators, which have multiple histone deacetylase (HDAC) inhibitory properties by suppressing the nuclear translocation of NFB and HDAC-3. In this study, nimbolide has a strong interaction with TNF-α. Moreover, nimbolide treatment increased superoxide dismutase (SOD-1), Nrf-2, GSH, and HO-1 protein expression and revoked LPS-triggered GSK-3β, TNF-α, p38 MAPK, mTOR, and protein expression (62). Recently, a peptide-conjugated liposome (iRGD-NIMLip) loaded with nimbolide was used to treat ARDS, a condition with many of the same symptoms as COVID-19, an anti-inflammatory phytomedicine (48). iRGD-NIML ip attenuated lipopolysaccharide-induced expression of inflammatory genes via PI3K/Akt/mTOR signaling pathway. In contrast to free nimbolide, iRGD-NIML ip also suppressed oxidative stress and cytokine storm. Consequently, these findings suggest that advanced drug delivery systems can be used as novel therapeutics for COVID-19 and other chronic respiratory diseases (48, 63). In addition, nimbolide displayed inhibitory effect on autophagy in an in vivo fibrosis model (64). Nimbolide treatment significantly reduced, mesenchymal and fibrotic markers, while epithelial markers were heavily upregulated. According to immunohistochemistry and confocal microscopy, nimbolide dampened LC-3 and p62 expression and increased Beclin 1 expression. Thus, nimbolide is suggested as a potent antifibrotic agent that regulates fibrosis-associated autophagy in this study (64). Extracellular matrix (ECM) deposition can be suppressed by nimbolide because it strongly interferes with the transforming growth factor (TGF-β)/Smad signaling axis and the ensuing epithelial-mesenchymal transition (EMT) process. Nimbolide showed both anti-inflammatory and antifibrotic effects, which significantly reversed existing inflammation associated with fibrosis. A collagen cross-linker called LOXL2 is abnormally expressed. Nimbolide was found to reduce these levels. The antifibrotic effects of nimbolide are significant (65). Nimbolide is a terpenoid chemical used to treat BLM-induced PF mice and TGF-β-stimulated cells by regulating autophagy via suppression of the pathway involved in EMT. The expression of mesenchymal and fibrotic markers is suppressed by nimbolide therapy, while epithelial markers are upregulated. Autophagy is controlled by nimbolide, which upregulates the level of Beclin-1 and downregulates protein of microtubules such as 1A/1B-light chain 3 and expression of p-62 (64).

Nimbolide treatment significantly decreased the infiltration of inflammatory cells, such as macrophages and neutrophils in bronchoalveolar lavage fluid (BALF). As well as reducing ROS in BALF. Nimbolide also decreased neutrophil elastase activity. Nimbolide reduced the recruitment of inflammatory cells and MCP-1 expression via inhibiting nuclear factor (NF-kB) and inhibitor of NF-kB (IB) phosphorylation in smoking- and LPS-induced pulmonary inflammation mice models (66). In addition, nimbolide significantly inhibited ERK and JNK activation in mice exposed to CS and LPS in the lungs were similarly inhibited by nimbolide in mice exposed to smoking and LPS (66, 67).

## Protective effects of nimbolide with different mechanisms in different organs

### Hepatoprotective activity of nimbolide

Chronic liver fibrosis occurs when liver cells are stimulated by physical, biological, or microbial stimuli for a long time (68). Inflammatory lesions and structural changes are also visible, as well as abnormal fibroblast accumulation and excessive ECM deposition (69). Liver cirrhosis and hepatocellular carcinoma (HCC) are the consequences of hepatic fibrosis. Hepatic fibrosis is a repair mechanism for chronic liver diseases, caused by hepatic stellate cells (HSCs) (70). In tribal medicine, nimbolide is used to treat liver disease by preserving the structural integrity of the hepatocellular membrane by reducing levels of hepatic markers. Nimbolide protects the hepatocellular membrane from damage caused by CCl4 (71). Nimbolide inhibits HDAC3 expression in autoimmune hepatitis (AIH) mice’s liver and AML12 cells to inhibit inflammation (42). Furthermore, long-term administration demonstrated significant histopathological protection of both liver and kidney without causing any adverse effects on liver and kidney (72). In this context, nimbolide showed hepatoprotective activity against CCl4-induced liver damage in a dose dependent manner in rats similar to that of Silymarin, as revealed by histopathological and transmission electron microscopic analysis (13).

Nimbolide showed to regulate the ROS redox-homeostatic imbalance and the immune modulatory response after Ochratoxin A (OTA)-induced acute liver inflammation in mouse models. The anti-inflammatory effect of nimbolide was found to be mediated by inhibiting cytokine production and decreased oxidative stress, via regulating protein peroxidation, protein expression of SIRT1-mediated pathways and protein carbonyl content (PC) (73). In addition, nimbolide can prevent cisplatin-induced hepatotoxicity and nephrotoxicity (74). Nimbolide may reduce inflammation in mice with HCC via inhibiting the NF-B pathway. From tumor initiation to tumor growth and metastasis, inflammation plays a significant role in the pathophysiology of cancer. Cirrhotic patients with a preexisting inflammatory condition had a 70% increased risk of developing HCC (75). During chronic inflammation, the pro-inflammatory cytokines such as TNF-α and IL-1β are released from hepatic Kupffer cells via NF-kB (75). HCC mice have a hyper inflammatory state in the tissue of hepatic system, as evidenced by elevated protein production of NF-kB and its down cascade effector proinflammatory cytokines IL-1β and TNF-α. When given to cell lines or an animal model, nimbolide reduces NF-kB signaling and inflammatory cytokines (31). By blocking NFk-B signaling and the Wnt/β-catenin pathway, nimbolide prevents cancer from spreading in HepG2 cells (76). Nimbolide therapy suppressed TNF-α, NF-kB, and IL-1 protein expression in HCC mice. Nimbolide also interacted hydrophobically with amino acid residues in the cavity of ligand binding in NF-kB and TNF-α, as determined by the molecular docking investigations. According to these findings, nimbolide reduced inflammation and inhibited NF-kB activation in mice with HCC generated by Diethylnitrosamine (DEN) and N-nitrosomorpholine (NMOR). Since NFk-B signaling is thought to be a key mechanism connecting inflammation in HCC, it is hypothesised that nimbolide may regulate cell proliferation by inhibiting this pathway (77, 78). AIH is a chronic liver condition characterized by extreme level of inflammatory markers. In controlling the activity of genes involved in inflammatory, HDAC3 plays a pivotal role. Some research suggests that nimbolide can prevent liver damage in people with AIH. Nimbolide treatment resulted in a reduction of the inflammatory cytokines IL-6, IL-1β, and TNF-α, as well as inflammatory cellular signaling molecules IkB-α, STAT3, and NF-kB. Nimbolide blocks HDAC3 expression, reducing inflammation in the liver of AIH mice and in AML12 cells (42). Glutathione peroxidase, catalase (CAT), concentration were all found to be up, while malondialdehyde and nitric oxide levels were shown to be significantly reduced by nimbolide. As a result, nimbolide’s antioxidant action and ability to promote apoptosis raise the possibility that they reduce lipid peroxidation (72).

### Anti-diabetic effect of nimbolide in diabetes

**Fig. 3:**
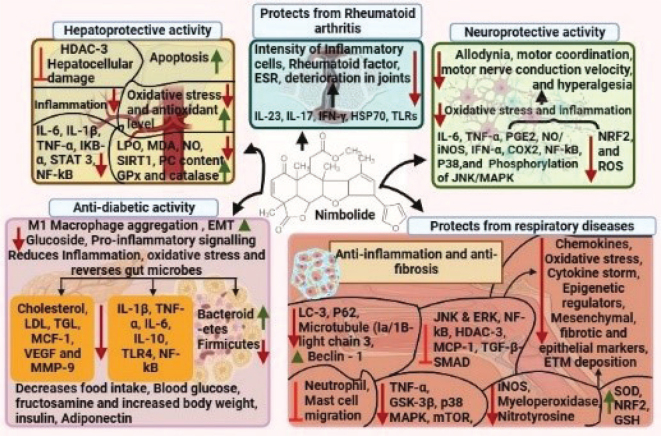
The protective mechanism of nimbolide (Image created from biorender.com).

The metabolic disorder diabetes mellitus (DM) is characterized by hyperglycemia and affecting more children and teens in developed and developing countries. Diabetic nephropathy, myopathy, and retinopathy are caused by the prolonged impediment of DM through changes in macro- and micro-vascular function (15, 79, 80). Several studies demonstrated the role of nimbolide as an effective treatment for diabetes. Nimbolide treatment effectively decreased food intake and blood glucose levels and improved bodyweight in STZ-induced animals. This effect is mediated by suppression of the levels of pro-inflammatory mediators, (cholesterol, TG, LDL, and HDL, MCP-1, VEGF, and MMP-9) and inhibiting the TLR4/NF-CB pathway in a very significant way (15) (Fig. 3). In pregnant diabetic rate model, nimbolide showed to reduce inflammation, oxidative stress, and to reverse gut microbiota, which protects them from gestational diabetes. Furthermore, nimbolide demonstrated a protective effect against streptozotocin-induced gestational DM in rats via alteration gut microbiota by stimulating the Bacteroidetes and inhibiting the firmicutes (14). Nimbolide therapy resulted in substantial reductions in both blood glucose and weight, as well as increases in both insulin and body mass index. Fructosamine and leptin were both greatly reduced by nimbolide, whereas adiponectin was significantly increased. Nimbolide treatment resulted in significant reductions in malonaldehyde (MDA) and total antioxidant capacity (TAC), as well as increases in CAT, SOD, glutathione S-transferase (GST) and glutathione peroxidase (GPx), and suppression of IL-1, TNF-α, IL-6, and elevation of IL-10 (14). The treatment of STZ-induced rats with nimbolide effectively reduced blood glucose and food intake, and increased body weight. The use of nimbolide significantly reduced the concentrations of pro-inflammatory mediators such as cholesterol, low-density lipoprotein, triglycerides, high-density lipoprotein, matrix metalloproteinase-9 and vascular endothelial growth factor. Nimbolide significantly suppressed the TLR4/NF-kB pathway (15). Due to its ability to block glucosidase, the nimbidiol is being studied as a possible anti-diabetic molecule. Elevated M1 macrophage aggregation, higher EMT and pro-inflammatory cytokines were all observed in diabetic kidney tissue. Functional deficiencies such as an increased resistive index, decreased blood flow in the renal cortex, and a lower glomerular filtration rate have been linked to the pathological alterations seen in diabetic mice. In addition, compared to wild-type (WT) mice, the diabetic kidney displayed elevated levels of p-Smad2/3, TGF-β1, p-P38, p-JNK and p-ERK1/2. Nimbidiol treatment reversed the effects to improve renal function in Akita mice by reducing inflammation, ECM buildup, and fibrosis. Nimbidiol protects the kidney from fibrosis and impairment in type 1 diabetes by reducing inflammation and ECM buildup, potentially through blocking TGF-β/Smad and MAPK signaling pathways (81).

### Anti-cancer effect of nimbolide

Nimbolide showed high cytotoxicity in various cancer cell lines. Nimbolide reported to inhibit cancer progression, via several mechanisms including prevention of oxidative DNA damage, pro-carcinogen activation and, increasing antioxidant activity, inducing apoptosis, inhibiting tumor cell proliferation, invasion, angiogenesis, and metastatic growth (31, 82–86).

Several studies have demonstrated the anti-cancer potential of nimbolide against breast cancer. In breast cancer xenograft models, nimbolide treatment inhibited proliferation of human breast cancer cells and reduced tumor mass and volume (85, 87). Under hypoxia, nimbolide increased prolyl hydroxylase activity and lowered cellular oxidative stress by upregulating RECK (88). Nimbolide reported to decrease cell proliferation, EMT, cell cycle progression, and migration, in breast cancer cells via downregulating the NF-κB pathway (89, 90).

This study investigates the potential anticancer activities of nimbolide in the context of pancreatic cancer. The data presented in our study demonstrate that nimbolide stimulates the overproduction of ROS, consequently modulating both autophagy and apoptosis in pancreatic cancer cells. The effects of autophagy inhibitors, specifically chloroquine diphosphate salt and 3-methyladenine, as well as the apoptosis inhibitor (z-VAD-fmk), were investigated. The impact of nimbolide-induced ROS generation on growth inhibition, metastasis suppression, and the underlying mechanisms involved. The findings indicated that nimbolide-induced ROS generation hindered cell proliferation by suppressing PI3K/AKT/mTOR and ERK signaling pathways. Additionally, it was observed that nimbolide-mediated ROS generation reduced EMT, migration, colony forming abilities and invasion, thereby inhibiting metastasis. These effects were attributed to mitochondrial-mediated cell death (apoptosis) rather than autophagy (91).

In addition, the use of nimbolide in combination with 5-FU showed a higher inhibitory rate in breast cancer than 5-FU alone (92). Cox-2, Bcl-2, and procaspase-3 expression was also decreased by nimbolide and P53 expression was increased (43, 93, 94).

In human Leukemia cell line, KBM-5, nimbolide showed to enhance the cytotoxic and apoptotic effects of TNF and chemotherapeutic drugs (5-fluorouracil, thalidomide) by inhibiting the IKKB-induced NF-κB pathway. Nimbolide targets IKK activation loop Cys179 to inhibit NF-κB. This effect was abolished by the addition of reducing agents and/or mutation of the Cys179 residue of IKK to alanine (95, 96). TRAIL and nimbolide treatment also induced cell death through ROS and ERK-mediated up-regulation of p53 and Bax, up-regulation of DR5 and DR4, and down-regulation of survival proteins, (97, 98). ERK1&2 gene silencing also blocked TRAIL-induced apoptosis and upregulation of DR5 and DR4 (47, 99). Interestingly, nimbolide synergized the effect of TRAIL to induce apoptosis in tumor cell lines, but not normal breast cells (100). A novel way of boosting anticancer effects may be found in the use of nimbolide as a sensitizer in combination regimens using TRAIL.

Activation of Wnt/-β catenin signaling pathway by NF-κB is one of the mechanism of carcinogenesis in various tumor types (101–105). GSK-β3 is a key component of the Wnt signaling pathway, which plays a role in the functional cross talk between NF-κB and Wnt/β-catenin. Free β-catenin is accumulated in the cytosol after GSK-β3 activation and eventually translocates to the nucleus, activating various tumor-related genes (106, 107). Interestingly, hepatic, breast and oral cancer treated with nimbolide have lower levels of GSK-β3 and β-catenin, which inhibits Wnt/β-catenin signaling (108–110). Nimbolide was also shown to inhibit cell proliferation through inhibition of IGF1/IGF1R/P13K/AKT pathway in prostate cancer cells as well as to induce apoptosis through activation of both intrinsic and extrinsic apoptotic pathways (110). Several studies reported the role of nimbolide, to suppress carcinoma cells viability and slows tumor growth by inhibiting CDK4/6 activity, (which leads to RB hypo phosphorylation and cell cycle arrest), and by inhibiting PI3K-AKT, MAP kinase, and JAK-STAT pathways (which are hyper activated in many types of carcinoma) (45, 109–115).

In cancer cells, NFκ-B, a transcription factor that promotes survival and counteracts apoptosis, is constitutively activated by inflammatory stimuli. Cancer cells rely on NF-κB for inflammation, proliferation, survival, immunity, differentiation, apoptosis, invasion, and metastasis. An inactive heterotrimer of the NF-κB proteins consists of p50, p65, and IκB when NF-κB is at rest in the cytoplasm. The IκB ubiquitinated at lysine residues 21 and 22, phosphorylated at serine residues 32 and 36, and degraded by the 26S proteasome in response to activation signals. In oncogenic progression, IB degradation allows the translocation of NF-κB heterodimer (p65, p50) to the nucleus for transcription of target genes (116–120). NFκB promotes tumor invasion, metastasis, and angiogenesis, which are major causes of cancer mortality and morbidity (121, 122). Nimbolide was reported to inhibit IκB degradation and prevents NF-κB nuclear translocation and thus causes apoptosis (96, 123). In addition, nimbolide suppressed the Wnt/β-catenin signaling pathway mediated by NF-κB in HCC and pancreatic cancer cells (124, 125). During embryonic development and tissue homeostasis, Wnt regulates the levels of co-activator β-catenin expression that controls the expression of key developmental genes. Wnt mutations cause cancer and birth defects in humans (121, 126). Nimbolide induced caspase-mediated apoptosis in prostate cancer by abrogating NF-κB and Wnt signaling. Additionally, nimbolide can significantly suppress the activation of oncogenic transcription factor STAT3. ROS are produced more when GSH/GSSG are imbalanced, which causes this effect (23, 41). A study showed that nimbolide prevented metastasis and tumor invasion by inhibiting PMA-induced phosphorylation of ERK1/2 and MMP-2/9 expression (46, 127). Taking together, it is evident that nimbolide inhibits tumor cell growth and migration by downregulating VEGF-A and MMP-2/9 expression, through the inhibition of ERK1/2, and by reducing nuclear translocations as well as the DNA-binding activities of NF-κB in cancer cells (127).

Checkpoints in normal cells regulate cell cycle progression through the sequential activation and inactivation of CDKs, which phosphorylate and dephosphorylate a number of regulatory proteins (128). In cancer, cell cycle defects and mutations in genes controlling the cell cycle are common, so agents that suppress tumor cell proliferation can be helpful (129). In this context, bladder cancer cells treated with nimbolide showed cell cycle disruption with a decrease in G0/G1 phase cells and an initial increase in S and G2/M phase cells as measured by flow cytometric analysis (130). Both the Chk2-p21WAF1-Cdc2/cyclin B1-Wee1 pathway and the Chk2-Cdc25C-Cdc2/cyclin B1-Wee1 pathway contributed to nimbolide-induced G2/M phase cell cycle arrest. In contrast to its effects on p38MAPK and AKT phosphorylation, nimbolide promoted JNK phosphorylation. MMP-9 activity was inhibited by nimbolide, which hampered wound healing migration and invasion. Lastly, nimbolide inhibited MMP-9 activity by inhibiting the binding activity of Sp-1, AP-1 and NFk-B motifs, all of which are important transcription factors. As a whole, the results suggest that nimbolide may be useful as a antineoplastic agent in the treatment of bladder cancer (130). The carcinogenic NF-kB (NF-kB) pathway can be inhibited by nimbolide as well. The pro-inflammatory transcription factor NF-kB plays a role in tumor initiation, oncogenic development, and resistance to apoptosis. In the cytoplasm, NF-kB is deactivated by forming complexes with I proteins such IKKα and IKKβ. Because of a potent nuclear export signal in I protein, this interaction predominantly localizes the inactive NF-kB complex in the cytoplasm. A malfunctioning gene or persistent activation of the IKK pathway that degrades both contribute to NF-nuclear B’s localization and constitutive activity in many cancer cells. This allows NF-kB to get access to the nucleus, where it can stimulate the production of many different genes that play a role in tumor development. Nimbolide was shown to prevent NF-kB from entering the nucleus and to slow down the breakdown of IB. The ensuing downregulation of several genes that regulate cellular growth led to cell cycle arrest. Apoptosis can be triggered by nimbolide by inhibiting nuclear factor kappa B. This resulted in the downregulation of Bcl-2 and the upregulation of cytochrome c, Bax, and Smac/DIABLO expression. In addition, nimbolide suppressed the Wnt/catenin signaling pathway that NFk-B had a role in HepG2 cells. Throughout development of embryo and tissue homeostasis, the Wnt family of glycoproteins modulates the quantity of the transcriptional co-activator catenin. Hence, abnormalities in the Wnt pathway are linked to cancer and congenital abnormalities in humans. Nimbolide induced caspase-mediated death via blocking NF-B and Wnt signaling (130). Nimbolide treatment showed to reduce the S and G2/M fraction with a reciprocal increase of cells in the subG1 fraction in dose-dependent manner. HT-29 cells exposed to nimbolide showed G2/M arrest accompanied by upregulation of cyclin D2, p21, chk2 and downregulation of cyclin A, cdk2, cyclin D1 and Rad17 (131). Exposure of HT-29 cells to more nimbolide for a longer period resulted in signs of apoptosis, including more sub-G1 cells, more annexin-V-positive cells, and more phosphotidylserine in the outer leaflets of mitochondria (132). Nimbolide exhibited dose-dependent inhibitory effects on HeLa cell viability by causing cell cycle arrest at G0/G1 phase with p53-dependent accumulation of p21. Nimbolide also downregulated cell cycle regulator proteins such as cyclin B, cyclin D1, and PCNA (133). Mice with HCC given nimbolide had their hepatic inflammation reduced, with expression of IL-1 beta, NF-kB, and TNF-α all going down, and expression of 4-hydroxynonenal going down as well, indicating that oxidative stress had been prevented. ZO-1, TNF-α, and NF-B are all targets of nimbolide’s interactions. Treatment of HCC mice with nimbolide reduced inflammation and oxidative stress and increased the expression of Tight Junction proteins. As a result, nimbolide shows promise as a natural therapeutic agent for the treatment of HCC; nevertheless, more research is needed in humans (78). Nimbolide is a limonoid extracted from the neem tree that has attracted a lot of study due to its powerful antiproliferative and apoptosis-inducing activities. Oral cancer cells treated with nimbolide exhibit a combination of apoptotic and autophagic phenotypes. Autophagy in SCC4 and SCC131 oral cancer cells and the exact point at which the phosphorylation state of PI3K and GSK-3 determines the decision between apoptosis and autophagy. Modulating the phosphorylation status of AKT and GSK-3 as well as the ncRNAs miR-126 and HOTAIR, nimbolide enhances apoptosis by counteracting the beneficial effects of cytoprotective in autophagy. There is an urgent need in cancer prevention and treatment for the advancement of phytochemicals such as nimbolide, which target the complex interactions between proteins and ncRNAs that govern the autophagy/apoptosis flux (134).

Epithelial tumor cells undergo phenotypic changes during metastatic progression, largely as a result of environmental stimuli, allowing them to adapt to the different microenvironments they encounter. A major player modulating these phenotypic conversions is the epithelial-to-mesenchymal transition (EMT). By inhibiting the TGF-β/Smad signaling axis and EMT, two major factors implicated in scleroderma, nimbolide administration induced regression of inflammation-driven fibrosis in a murine model of bleomycin-induced scleroderma. The level of collagen cross-linker, lysyl oxidase like-2, as well as the amount of ECM deposition was also reduced by nimbolide. There has been evidence that nimbolide regulates autophagy signaling in pulmonary fibrosis induced by TGF-1 in vitro and bleomycin in vivo. Mesenchymal and fibrotic markers were downregulated, whereas epithelial markers were upregulated. As a result of reducing collagen deposition, oxidative stress, and TGF – Smad and ECM protein expression, nimbolide prevented renal fibrogenesis and inflammation (Nagnin et al., 2021).

### Neuroprotective effects of nimbolide

Nimbolide demonstrated neuroprotective effects in animal models of neurodegenerative diseases. As a result of peripheral neuropathy (induced by partial sciatic nerve ligation), animal models with nimbolide treatment showed significant reductions in allodynia, motor coordination, motor nerve conduction velocity, and hyperalgesia (33, 135). In addition, nimbolide showed neuroprotective properties against cisplatin-induced neurotoxicity, where brain tissue was well preserved (136, 137). The results of this study evaluate the impact of nimbolide on neuro-inflammation in LPS-activated BV2 microglia. LPS-activated BV2 cells treated with nimbolide had significantly lower levels of IL6, TNF-α, PGE2, NO/iNOS, IFNα and COX-2. Further testing showed that nimbolide attenuated the LPS-induced upregulation of phospho-IB and phospho-p65 protein expression. Nimbolide also inhibited p38 and phosphorylation of JNK MAPK and LPS-induced acetylation of NF-kB, enhanced binding with consensus sites, and transactivation. Nimbolide inhibited ROS production in cells, and this action was accompanied by a rise in HO-1 and NQO-1 protein levels, two antioxidant enzymes. Nimbolide treatment of BV2 microglia to decrease cytoplasmic Nrf2 levels while increasing levels in the nucleus. The treatment with this chemical also raised IS luciferase activity and Nrf2 binding to ARE consensus sites. By mechanisms resulting in simultaneous inhibition of NF-kB and MAPK pathways, nimbolide decreases neuro-inflammation (BV2 microglia). The antioxidant activity of Nrf2 and the deacetylation activity of SIRT-1 are both thought to have a role in its anti-inflammatory effect (22).

### Nimbolide on targeting pathophysiological modules of arthritis

Nimbolide, a bioactive compound found in *A. indica*, has notable properties such as antioxidant, anti-inflammatory, anticancer and immune-modulatory activities. Limited research indicates that the administration of nimbolide has an impact on the reactions associated with rheumatoid arthritis. However, the precise molecular processes behind this phenomenon have yet to be thoroughly elucidated. The objective of this study is to investigate the impact of nimbolide on the modulation of toll-like receptors in order to mitigate the symptoms of rheumatoid arthritis. The administration of nimbolide resulted in a notable decrease in inflammatory cells, rheumatoid factor, and erythrocyte sedimentation rate (ESR), while also demonstrating an improvement in body weight. The findings of the study suggest that nimbolide has the ability to reduce the severity of rheumatoid arthritis by suppressing the expression levels of toll-like receptors, IL-23, IL-17, IFN-γ and HSP70. The administration of nimbolide resulted in a notable decrease in the intensity of inflammation and joint deterioration, demonstrating similar efficacy to piroxicam: a well-accepted anti-inflammatory and non-steroidal medicine commonly prescribed for the management of rheumatoid arthritis. Based on the available evidence, it is reasonable to assert that nimbolide exhibits promise as a viable contender for therapeutic intervention aimed at targeting the pathway (toll-like receptors) in the context of rheumatoid arthritis (138).

## Conclusion

A comprehensive look at the science behind nimbolide reveals that it possesses a number of biological roles. This makes it one of the most effective compounds for the prevention and treatment of a variety of chronic diseases. It also has tremendous potential for drug discovery. In spite of this, more clinical research is needed in order to support the findings that have been mentioned above. It should be noticed, however, that there is still a lot of potential for more powerful analogs and formulations of nimbolide to be developed. This will improve the safety and effectiveness of medicines designed for treating several chronic conditions. As such, thorough clinical evaluation and trials are necessary to determine the effectiveness and toxicity of nimbolide and its formulations. Once this is achieved, nimbolide has the potential to become a valuable medicinal drug.

## Data Availability

Not applicable.
